# Emotion recognition for human–computer interaction using high-level descriptors

**DOI:** 10.1038/s41598-024-59294-y

**Published:** 2024-05-27

**Authors:** Chaitanya Singla, Sukhdev Singh, Preeti Sharma, Nitin Mittal, Fikreselam Gared

**Affiliations:** 1 Chitkara University Institute of Engineering and Technology, Chitkara University, Punjab, India; 2 Department of Computer Science, Multani Mal Modi College, Patiala, Punjab India; 3 Skill Faculty of Engineering and Technology, Shri Vishwakarma Skill University, Palwal, Haryana 121102 India; 4 Faculty of Electrical and Computer Engineering, Bahir Dar University, Bahir Dar, Ethiopia

**Keywords:** Speech emotion recognition (SER), Deep learning, High-level features, Emotion recognition, Punjabi database, Punjabi speech emotion recognition, Health care, Engineering

## Abstract

Recent research has focused extensively on employing Deep Learning (DL) techniques, particularly Convolutional Neural Networks (CNN), for Speech Emotion Recognition (SER). This study addresses the burgeoning interest in leveraging DL for SER, specifically focusing on Punjabi language speakers. The paper presents a novel approach to constructing and preprocessing a labeled speech corpus using diverse social media sources. By utilizing spectrograms as the primary feature representation, the proposed algorithm effectively learns discriminative patterns for emotion recognition. The method is evaluated on a custom dataset derived from various Punjabi media sources, including films and web series. Results demonstrate that the proposed approach achieves an accuracy of 69%, surpassing traditional methods like decision trees, Naïve Bayes, and random forests, which achieved accuracies of 49%, 52%, and 61% respectively. Thus, the proposed method improves accuracy in recognizing emotions from Punjabi speech signals.

## Introduction

Speech Emotion Recognition (SER) is a prominent research area in human–machine interactions. It is a way to capture and analyse human emotions to understand a person’s mental state. SER is considered to enhance the effectiveness of speech recognition systems^1^, making it valuable in various fields such as criminal investigation, intelligent assistance, healthcare systems^2^, surveillance, and detection of potentially dangerous situations^3,4^. This technology enables the machine to detect human emotion in different scenarios such as in crowded environments, where SER can be a valuable tool for automatically analyzing physical interactions of individuals that seems to be difficult manually.

**Figure 1:**
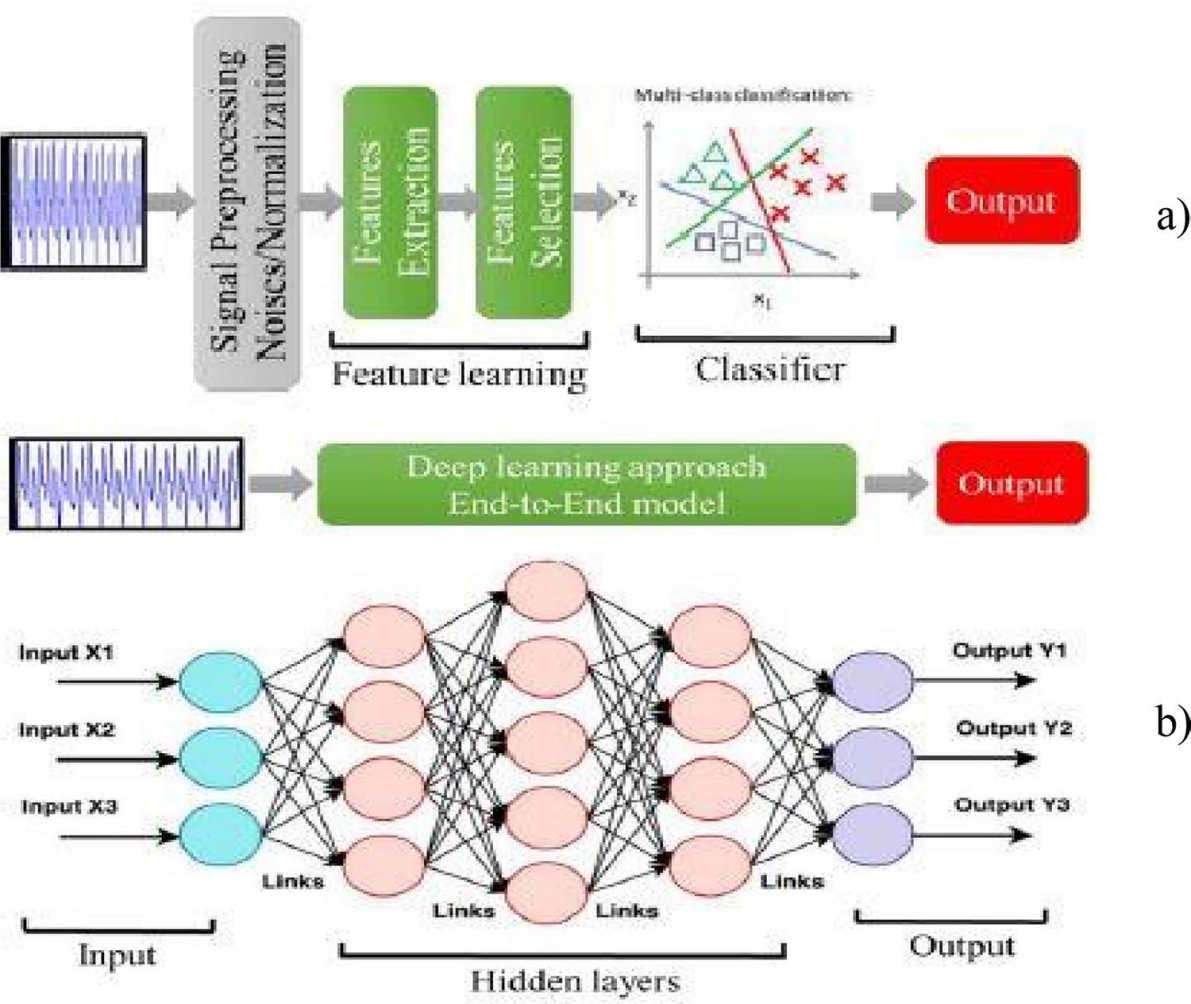
(**a**) Traditional Approach for SER (**b**) DNN Based Approach for SER.

In SER, discrete emotions^5^ and emotional dimensions^6^ are two conceptual paradigms. Discrete emotions consist of happy, sad, neutral, and angry whereas emotion dimensions contain vocals, audio, arousal, dominance, and many more. Moreover, the two major components of SER are linguistic and paralinguistic where linguistic defines what has been said? and paralinguistic is how it has been said? respectively. Paralinguistic is an emerging research area with major challenges because of its language barrier. However, various researchers worked on different languages such as English, Hindi, Urdu, and many more^7–10^. These languages have defined datasets, which are publicly available as shown in Table 1. Furthermore, the dataset needs to be processed to capture dynamic information of the speech signals to analyse human emotions. Deep learning models and spectrograms are used to process this data to obtain feature maps. The task involves the extraction of efficient speech features. Extracting intrinsic features from raw speech data and converting them into suitable formats for further processing is a persistent challenge in speech emotion recognition. The traditional method for extracting the speech signal consists of two parts; (i) pre-processing the signal and (ii) extracting the features. The extracted features are used for emotion classification. In Fig. 1, the traditional and deep learning models are shown that help to differentiate the working of both methods. The combination of CNN and spectrogram work has proven various advantages over traditional methods. These studies identified single-channel spectrograms and multi-channel spectrograms^11^. In a single-channel spectrogram, short-time Fourier transformation is applied to audio signals, and the resulting spectrogram is fed into the potential CNN model. In contrast, multi-channel spectrograms are defined as the extraction of multiple spectrograms for each audio signal and then input in the model.

**Table 1:** Overview of various datasets.

Dataset	Language	Size	Emotions	Type	Modalities	Access Type
Berlin Emotional Database (EmoDB) ^12^	German	7 Emotions × 10 speakers (5male, 5female) × 10 utterances	Anger, boredom, disgust, fear, happiness, sadness, neutral	Acted	Audio	Freely Available
Chinese Emotional Speech Corpus (CASIA) ^13^	Mandarin	6 Emotions × 4 Speakers (2male, 2female) × 500utterances (300parallel, 200 non-parallel texts)	Surprise, happiness, sadness, anger, fear, neutral	Spontaneous	Audio	Commercially Available
The Interactive Emotional Dyadic Motion Capture Database (IEMOCAP) ^7^	English	10 speakers (5male, 5 female)1150 utterances	Happiness, anger, sadness, frustration, neutral	Acted	Audio, Visual	Free to research use
Toronto Emotional Speech Database (TESS) ^14^	English	2 speakers(female), 2800 utterances	Anger, disgust, neutral fear, happiness, sadness, pleasant, surprise	Acted	Audio	Freely Available
Chinese Annotated Spontaneous Speech Corpus (CASS) ^15^	Mandarin	7 speakers (2male, 5 female), 6 h of speech	Anger, fear, happiness, sadness, surprise, neutral	Spontaneous	Audio	Commercially Available
Chinese Natural Emotional Audio–Visual Database (CHEAVD) ^16^	Mandarin	238 speakers 140-min emotional segments from movies, and TV- shows	Anger, anxiety, disgust, happiness, neutral, sadness, surprise, and worried	Spontaneous	Audio, Visual	Free to research use
Danish Emotional Speech Database (DES) ^17^	Danish	4 speakers (2male, 2 female)10 min of speech	Neutral, surprise, anger, happiness, sadness	Acted	Audio	Freely Available
eNTERFACE’0 5 Audio-Visual Emotion Database ^18^	English	42 speakers (34male, 8 female) from 14 nationalities, 1116 videos sequences	Anger, disgust, fear, happiness, sadness, surprise	Elicited	Audio, Visual	Freely Available
SUSTBangla Emotional Speech Corpus (SUBESCO) ^9^	Bangla	20 Professional actors (10 males, 10 females) participated in the recording of 10 sentences that consists of 7000 utterances	Anger, Disgust, Fear, Happiness, Neutral, Sadness and Surprise	Acted	Audio	Freely Available
Urdu-Sindhi speech emotion corpus ^10^	Urdu, Sindhi	1435 utterances	Happiness, anger, sadness, disgust, surprise, sarcasm, neutral	Acted	Audio	For Research use available on Request
Indian Institute of Technology Kharagpur Simulated Emotion Hindi Speech Corpus (IITKGP- SEHSC) ^8^	Hindi	12,000 utterances by 10 actors	Happy, anger, fear, disgust, surprise, sad, sarcastic, neutral	Acted	Audio	For Research use available on Request

India is a multilinguist country where most Indians live in rural areas. It has been determined that with time, various languages have been demolished and others are endangered. In this study, it has been identified that there are 1652 mother tongues in India including 103 foreign languages. As per the Indian constitution, there are 22 major languages in India out of which Punjabi is one of the widely spoken languages^19^ The distribution of Punjabi language around the globe for the top three countries are 48.2%, 2.8%, and 1.5% in Pakistan, India, and Canada respectively^20^**.** Additionally, it has been observed that there is limited work available for speech emotion detection using the Punjabi language. Another shortcoming is the non-availability of public datasets in the Punjabi language. To address this issue a novel dataset is created by the researcher.

**Figure 2:**
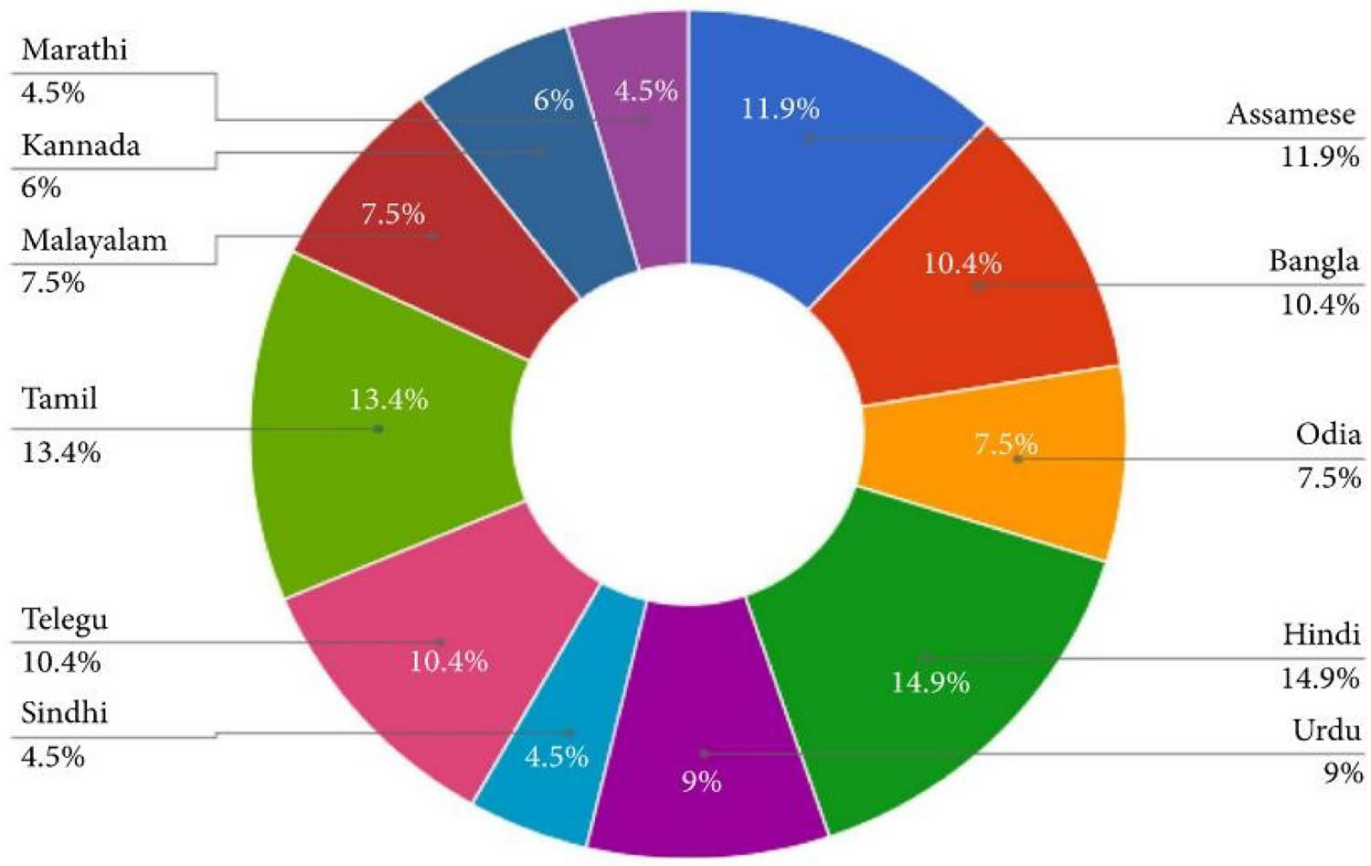
Analysis of experiments done on Indian languages for speech emotion recognition in the last two decades.

It is analyzed that there is no prior standardized multimodal emotion dataset, which contains recordings of speech and text of people who speak native languages in the Punjabi Language. Figure 2 shows an analysis of research works done for some of the Indian languages in the last two decades^21^.

The PEMO dataset^22^ is created to solve the issue. This dataset consists of web series and movies from YouTube. Further, every stream of the taken utterance is divided into smaller segments. This helps to cover the sample of speech of one speaker with minimum background noise. These utterances are labeled by three annotators on a 5-point scale namely happy, angry, sad, neutral, and none of the mentioned. These utterances were taken from native Punjabi speakers having no hearing loss or mental issues. The final label selected for the utterance depends on the common label used by all annotators. However, the utterances that do not have common labels were removed from the dataset. It has been identified that a neutral label has the maximum number of utterances, whereas a sad label is labeled with the minimum number of utterances.

The role of the PEMO dataset^22^ is to recognize emotions effectively. Python coding is used to convert audio signals to spectrograms. Further, these resulting spectrogram signals are input into (CNN) network to train the model. In the end, the trained model is tested on a 20% dataset that helps to recognize human emotions.

Therefore, the main contribution of this paper is speaker-independent emotion recognition which is summarized below.

1. The emotion-labeled speech corpus is expanded for the Punjabi language by utilizing freely available resources from multiple social media platforms. The objective involves the systematic collection of a diverse and extensive dataset of spoken Punjabi language that is appropriately labeled. The process entails conducting targeted searches and applying filters and keywords to gather relevant speech data. The collected data will then undergo an annotation process to accurately label the expressed emotions in each speech sample, categorizing them into four fields: happy, sad, angry, and neutral.
2. The SER plays a crucial role in developing a system capable of recognizing emotions for Punjabi speakers using speech signals in uncontrolled environments. In uncontrolled environments, the acoustic characteristics of speech signals can be influenced by various factors such as background noise, different recording devices, varying speaking styles, and environmental conditions. These factors pose significant challenges for emotion recognition systems as they can affect the accuracy and robustness of the recognition process. To address these challenges domain adaptation and noise reduction techniques are used.
3. To address the challenges associated with the uncontrolled environment for selecting robust features in emotion recognition, spectrograms have been utilized. Spectrograms capture the time-varying spectral information of speech signals, providing a comprehensive representation of emotional content. They are robust to noise and variations in recording conditions, allowing for more accurate and reliable feature extraction. Moreover, spectrograms offer valuable visualizations that aid in the interpretation and analysis of emotional cues. By leveraging spectrograms, researchers can develop more effective and robust emotion recognition systems that are capable of accurately recognizing emotions from speech signals in various real-world scenarios.

Organization of the research article: The article is structured as follows: Sect. “Background” provides a comprehensive review of current methodologies in SER, highlighting their advantages and disadvantages. Section “Proposed methodology” presents the proposed method, outlining the approach taken in this research. In Sect. “Experiment & results”, the experimental data and results are discussed in detail. Finally, Sect. “Conclusion and future work” explores potential future research directions that can be pursued based on the findings of this study.

## Background

The typical SER consists of two major parts (1) a unit that is processed in nature and retrieves the best feature vectors from signals of voice, and (2) using its feature vectors, the classifier can detect the hidden emotions in speech. The details of feature extraction and categorization methods are provided in this section. The selection of speech vectors is one of the most prevalent issues faced by systems of SER that permit an easy distinction between different emotions. The variations in various speakers, styles of speaking, or speaking rates, as well as different sentences directly impact the extracted features like the energy contour and pitch^23–26^. Breaking down voice signals into smaller components is one method known as frames. Global features are extracted from complete utterances whereas local features are extracted from each frame thus global features result in smaller dimensionality features, minimizing the amount of computing required. Furthermore, it's possible that a spoken utterance can be associated with multiple emotions; each emotion is associated with distinct frames. Moreover, the process of identifying boundaries between these frames is difficult due to the expression of certain emotions that vary between speakers as well as cultural differences and changes in the environment. Most of the research in the literature was done in a monolingual emotion classification context, with no consideration for cultural differences across speakers. The method was created to extract features that are advanced by using magnitude spectra and then making a comparison with the features that are hand-crafted. The author used a single context-dependent DNN with several voice tasks to alter a variety of Gaussian mixtures^27^. This author devised a system for analyzing the back-and-forth utterance levels of speech and then predicting the emotion of a speech using the results^28^. A few algorithms have been employed to elicit SER^29,30^. However, research has revealed that every classifier is dependent on the domain while comparing in case of accuracy as well as data quality. An aggregated approach that includes many classifiers is also being researched for enhancing SER accuracy^31^.

As technology rapidly adapts to processes from beginning to conclusion for classifying tasks with the use of algorithms based on deep learning. It is becoming increasingly important to research hierarchical systems to conduct SER for exceedingly difficult data sets. The automated process of extraction of discriminative features, which enables effective categorization of many types of data, is one of the strengths of the learning method which is known as end-to-end. The author pioneered learning based on features in SER by employing CNN to learn certain salient that affect the user^32^. They employed publicly available speech databases containing diverse languages. In terms of speaker variation, variation in language, and noise in the environment they were able to achieve high-quality results using learned features in comparison to other known feature representations. There are many approaches for employing CNNs to recognize emotion, but some use spectrograms to identify emotions in speech, which is the first stage in the process of SER. The spectrogram-based SER approaches have included an additional classifier on fully linked layers to increase the processing capability of the model. In this, the researcher contributes the feature block formed from the feature vector's effect salient is further supplied to an SVM classifier to determine the emotion class of a voice utterance^32^. Another point of reason that differentiates our work from previous studies is that we've implemented augmentation along with early stopping of the model to prevent overfitting of the model. This helps in better training of the model as it solves two problems, one is data scarcity, and the other is overfitting of the network model. In comparison to other architectures, the proposed method is less susceptible to overfitting when using limited data for training. Table 2 shows the summary of the work done by various researchers based on the classifiers used.

**Table 2:** Overview of literature review based on classifiers.

References	Dataset	Emotions	Classifier	Result Analysis
Yoon et al. ^33^	IEMOCAP	Happy, Sad, Angry, Neutral	RNN	A multimodal dataset was used taking speech and text modality. The accuracy achieved by combining both modalities was 68.8%
Kumbhar et al.^34^	RAVDEES	Happy, Sad, Angry, Surprise, Disgust, Fear	LSTM	80.81% accuracy was achieved by extracting the MFCC features after applying the LSTM model to the dataset
Mustaqueen et al. ^35^	IEMOCAP	Happy, Sad, Angry, and Neutral	CNN	72.2% accuracy was achieved on the IEMOCAP dataset. A novel SER architecture for the SER was also created
Shixin et al. ^36^	IEMPCAP, MELD	Happy, Sad, Angry, and Neutral	LSTM	63.64% accuracy was achieved on the MELD database. Spectrograms are generated from the audio data and word embedding is generated from the Text data using Word2Vec and the autoencoder fusion method is used for the fusion of both modalities
Makiuchi et al.^37^	IEMOCAP	Happy, Sad, Angry, and Neutral	CNN, Word2Vec	73% accuracy was achieved using score-based fusion on the IEMOCAP dataset. 70% accuracy was achieved on the speech modality by extracting the spectrograms from the signals
Padi et al. ^38^	IEMOCAP	Happy, Sad, Angry, and Neutral	ResNet, BERT	Score Based fusion method was used for the recognition of emotions from multimodalities i.e., speech and text. After fusing the overall accuracy of the system was 75.76%
Yenigalla et al. ^39^	IEMOCAP	Happy, Sad, Angry, and Neutral	CNN	68.5% accuracy achieved. The spectrograms are extracted from the speech signals and fed into the model for the recognition of emotions
Khan ^40^,	Cross-Corpus Datasets	Happy, Sad, Angry, and Neutral	Machine Learning Algorithms	The system uses cross-corpus to train the machine learning models. MFCC features are extracted from the speech signals. The model gives 91.25% accuracy based on the XGBoost classifier on the URDU dataset

It has been observed from the literature that only a few datasets are available in Indian Languages. Some of the Indian languages in which the datasets are available are Hindi^8^, and Urdu^10^. There is no ‘spontaneous audio dataset’ publically available for the Punjabi Language. So, there is a need to develop a labeled Punjabi Speech Emotion Database to train the machine for emotion recognition.

## Proposed methodology

**Figure 3:**
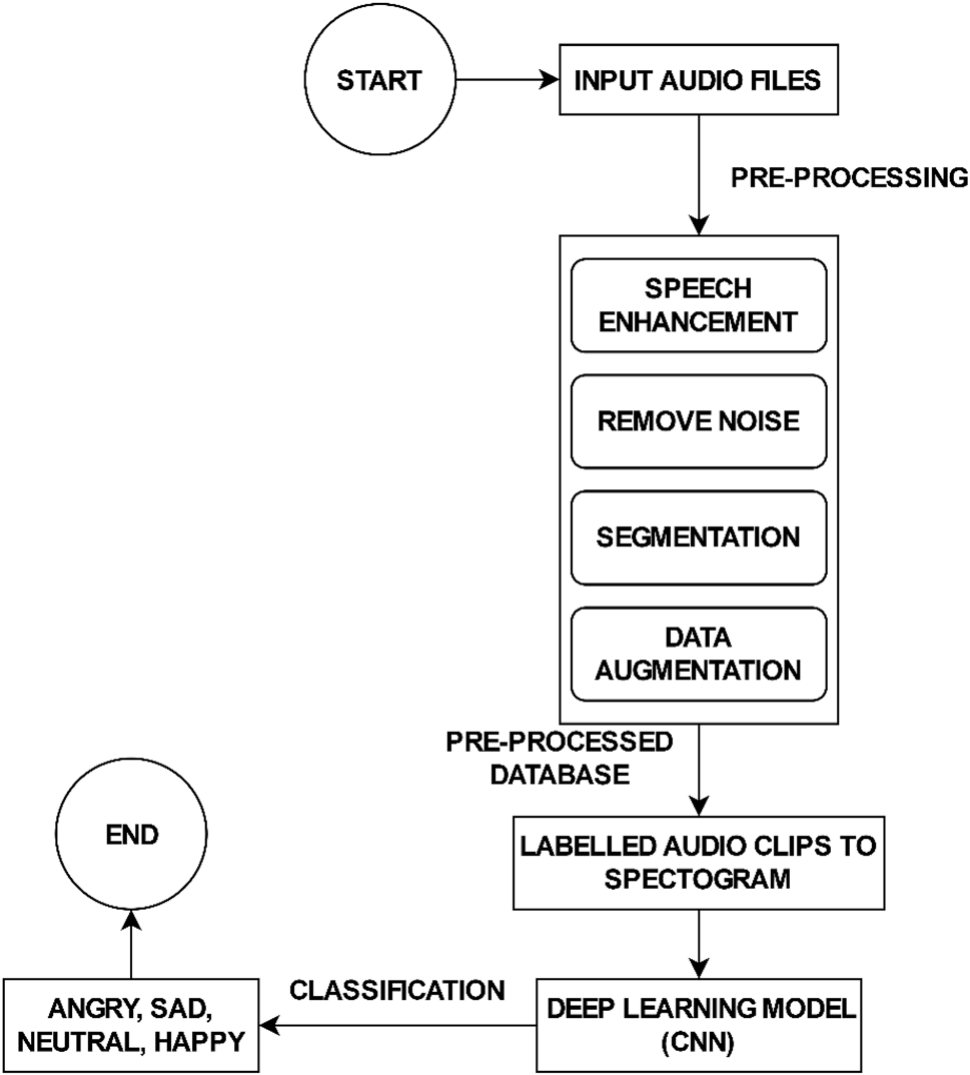
Flowchart for Speech Emotion recognition system.

The specified system uses a technique (learning) based on features that are driven by a discriminative CNN and spectrograms for detecting the speaker’s state related to emotions. In Fig. 3, the flowchart of the proposed methodology is presented. In the beginning, the audio files are considered as input and pre-processing has been done on the raw data files. These data files are taken from different sources. Once the raw data is collected speech enhancement is done by increasing the speech quality, and intensity using the PRAAT tool. In the next step, unwanted parts of the audio clip have been removed, for example, noise, and silence using noise reduction techniques. To categorize audio clips into four classes, segmentation has been done where the audio clip is divided into smaller parts to get the single emotional voice of a single person. Furthermore, for the count balance of four classes, data augmentation is done that removes the biases of the selected categories. The output of the pre-processing is the labeled audio clips contained in the pre-processed database. Moreover, this pre-processed database is converted into spectrograms so that it can be fed into deep-learning models. In addition, the model extracts the spectral features from the spectrogram that will be used for the training of the machine.

The essential components of the proposed framework have been detailed in the following sections.

### Retrieving spectrograms from voice

**Figure 4:**
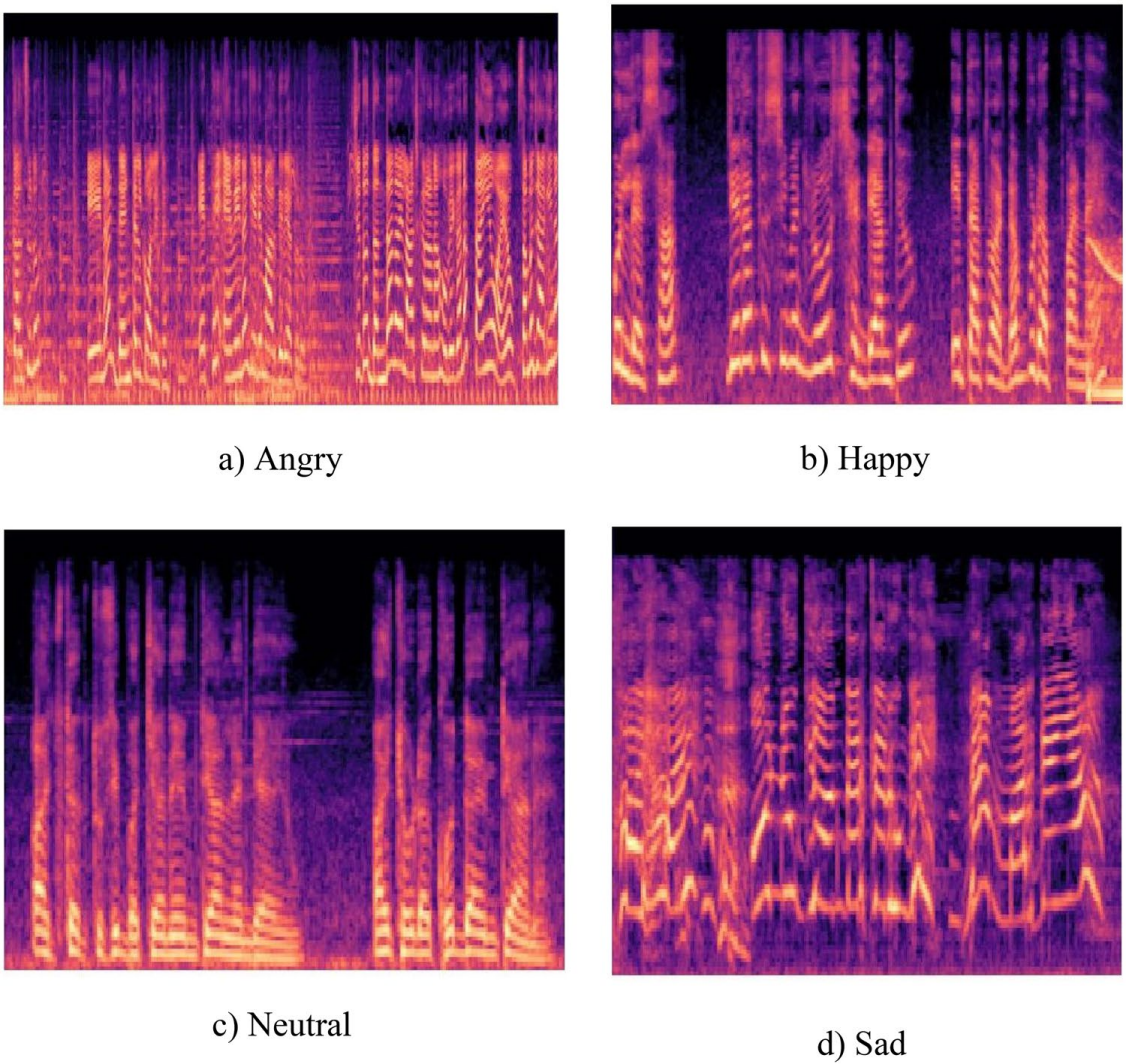
Spectrograms of various emotions.

A representation of the volume or strength of a signal across an individual waveform is called the spectrogram. By analyzing the strength of energy in a specific region it is also possible to observe the variations in energy over time. Generally, spectrograms can be brought into use for detecting the frequencies of the continuous signal. It’s a 2-D graphical representation in which the horizontal line denotes time, and the vertical axis denotes frequency. The strength of any particular frequency component f over the given moment, that is t inside the spoken signal can be described by the darkness or color of the spot S in the spectrogram (t, f). Figure 4. shows the spectrograms of one audio file taken from each emotion category. The spectrogram for angry emotion is shown in Fig. 4a, whereas for happy, neutral, and sad emotions is shown in Fig. 4b, c, d.

### Convolutional neural network

Convolutional neural networks (CNNs) are sophisticated models that have led to breakthroughs in the field of image categorization^26,41,42^. By applying the series of filters to the original data of each pixel in the picture, CNNs learn and extract the most important properties. The model then constructs categorization using these properties. Fully connected layers, extract feature that maps while concurrently categorizing the derived features, and pooling layers, which lower the dimensions of feature maps and thus decrease process time. The layers are normally put in a logical order, with the number of convolutional layers employed first, then pooling layers, and lastly fully connected layers.

The term "cluster" is most associated with a CNN, which is a hierarchical neural system composed of a succession of convolutional layers and pooling layers that perform feature extraction by layer-by-layer transforming images (including a spectrogram) to a higher abstraction. The first level comprises basic features like edges and raw pixels, whereas the subsequent layers have local discriminative characteristics, and the final dense (fully joined) layer creates an overall representation of the native convolutional features that is later supplied to the machine learning algorithm. Every convolutional kernel produces a feature map with activation values corresponding to the existence of certain properties. Many feature maps are created at each layer of convolution. The pooling layer is used to avoid overfitting and reduce computations inside the network. Max pooling is the most common pooling approach that keeps the most valuable value while discarding all other values found in the area. Fully linked layers utilize larger filters to address more intricate characteristics of input layers. The effectiveness of these models is determined by the choice of proper kernel size and shapes, as well as neighborhood pooling.

### Proposed model architecture

**Figure 5:**
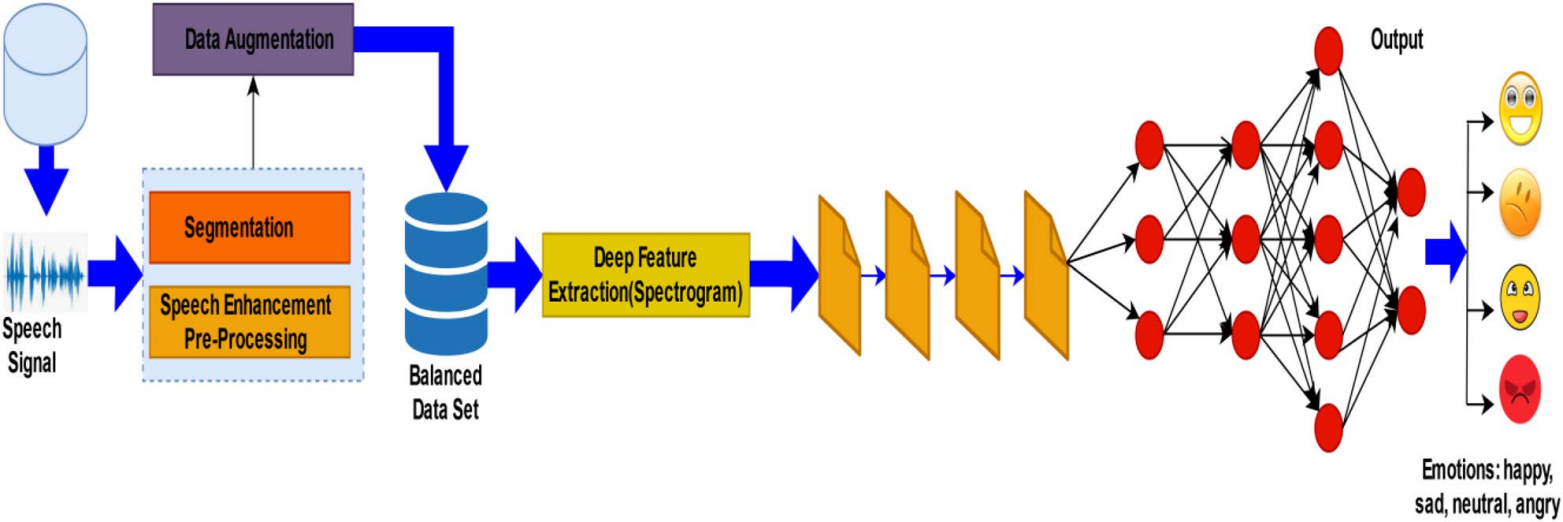
Methodology for Emotion Recognition.

As shown in Fig. 5, the speech signals that act as input to the model are collected from the various multimedia sites. These signals are processed and segmented using the PRAAT software. The segmentation of the speech signals is done in such a way that each utterance or file consists of a single emotion by a single person. The person may be of any gender. The files taken from various movies, and plays, the utterances taken consist of some background noise. These utterances are labeled with 4 emotions, (1) happy, (2) sad, (3) angry, and (4) neutral. Out of 70% of selected audio files, 50% are selected from neutral having the highest label whereas 10% is the lowest for sad audio. However, happy, and angry contain 18% and 22% of the dataset respectively.

The collected dataset is very unbalanced as several utterances for one emotion are more than another emotion category. To balance the dataset for proper training of the model data augmentation technique is applied. The time masking technique is used to augment the speech signals.

**Figure 6:**
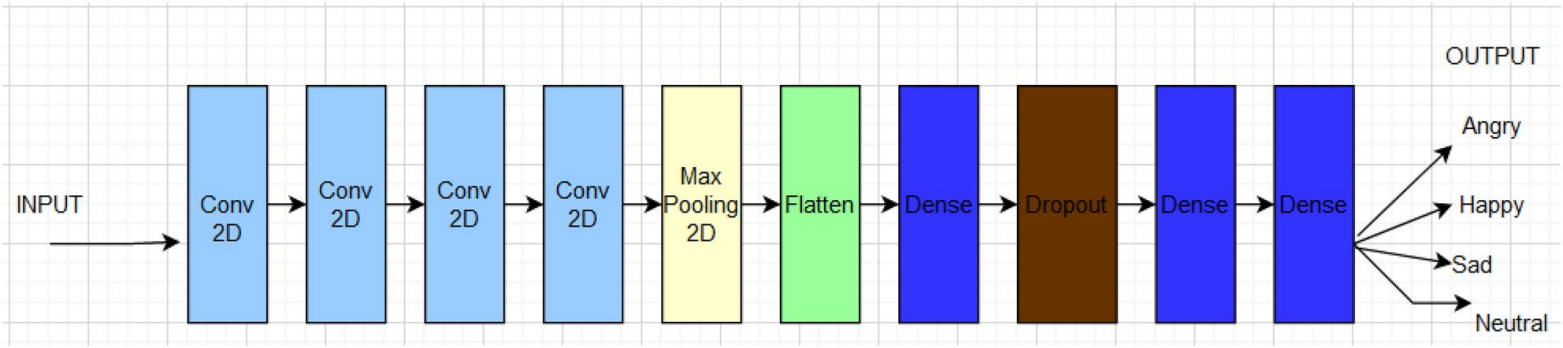
CNN Architecture.

The suggested CNN architecture consists of an input layer, 4 convolutional layers, 1 pooling layer, 3 dense layers, and a dropout layer as shown in the CNN architecture Fig. 6. CNN receives spectrograms that have been derived from emotive sounds. These spectrograms are normalized and only fed to the network. The spectrograms were 16 × 224 pixels in size. After that, they were resized to 224 × 224 pixels for use in CNN. Convolutional kernels are widely used as the input in the early layer levels for extracting feature maps.

**Figure 7:**
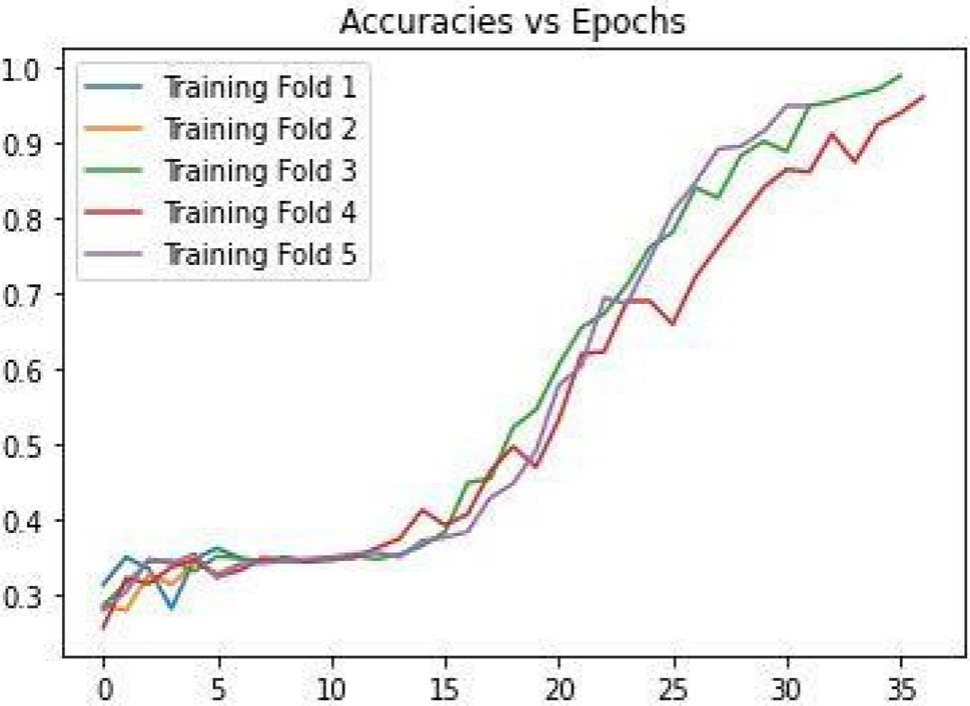
Accuracy’s versus Epochs.

The motive is to train and verify models. These spectrograms were split in the ratio of 80:20, whereas 80% was used for training and 20% for confirming the model's performance. In this paper, a fivefold cross-validation procedure was adopted. The training accuracy against the number of epochs for the fivefold validation process is shown in Fig. 7. It depicts that with the increase in epochs, the training accuracy also increases. In addition, when there are a smaller number of epochs the accuracy for the training fold 5 is also less but as the number of epochs increases, the accuracy for the training fold 5 is highest.

**Figure 8:**
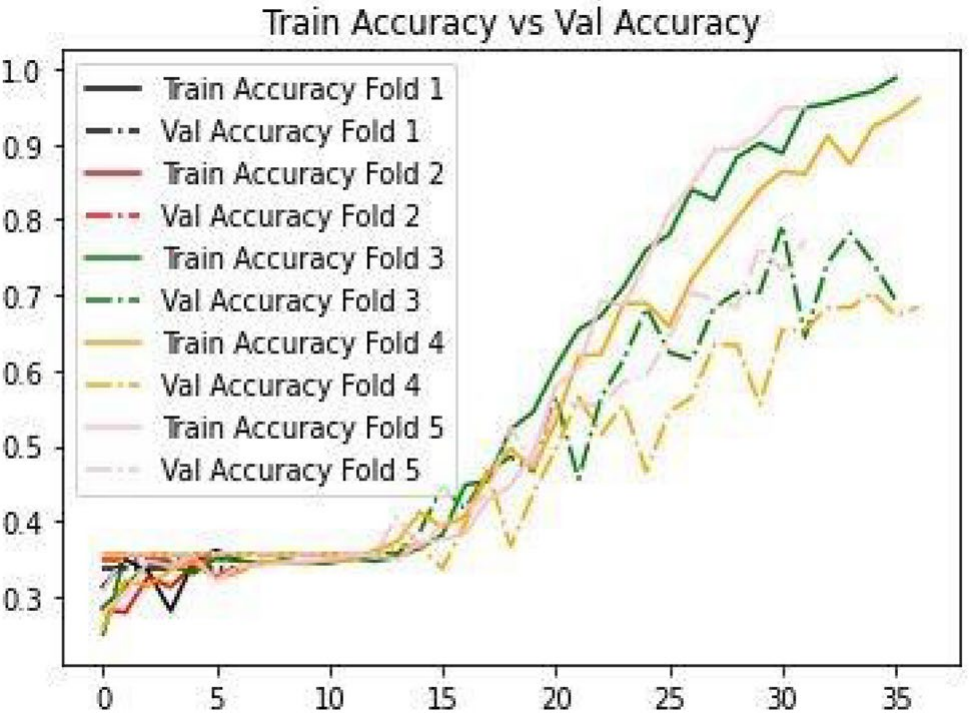
Train Accuracy versus Val Accuracy.

In this, the author takes the best model which has the highest accuracy from the 5 folds. The accuracies for all the folds in the fivefold validation are displayed in Fig. 8. By that, the training accuracy is more than the validation accuracy in all five folds. In every fold, the test size is 20 percent of the total dataset, and the rest is used for training and validation purposes. Every time a random set of features is selected and passed to the model for training and validation purposes. The best model with the highest training accuracy (69%) was achieved using 50 Epochs and early stopping criteria so that the model cannot be overtrained.

**Figure 9:**
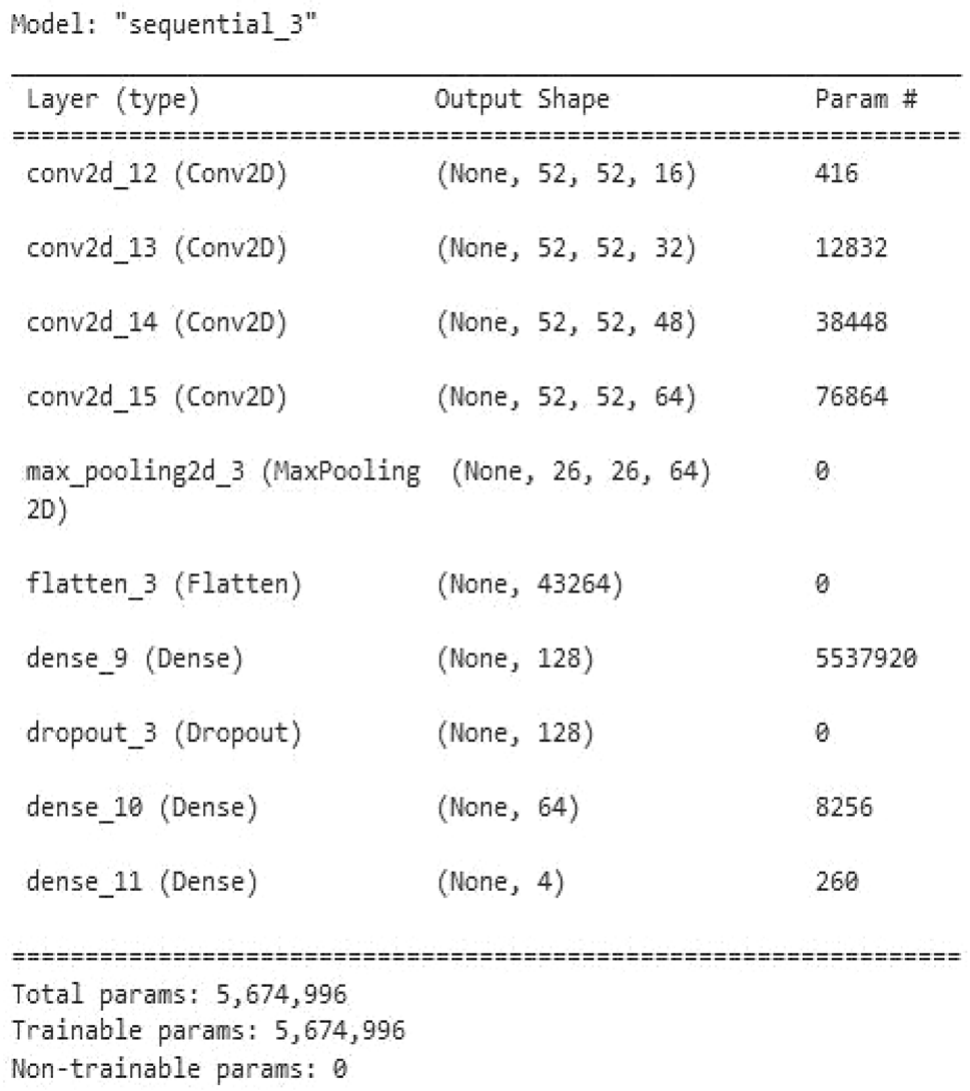
Proposed CNN Model.

The total number of trainable parameters is 5,674,996 and 0 non-trainable parameters. The implemented CNN model is shown in Fig. 9. is given below.

### Ethical approval

All procedures followed were in accordance with the ethical standards of the responsible committee on human experimentation (institutional and national).

### Human and animal rights

This article does not contain any studies with human or animal subjects performed by the any of the authors.

## Experiment & results

The experiment aims to develop a Speech Emotion Recognition (SER) system for the Punjabi language using deep Convolutional Neural Networks (CNNs). First, a dataset was meticulously assembled by manually collecting audio files from multimedia platforms like YouTube. These files, encompassing novel utterances and short clips, were then expertly labeled by Punjabi language experts, ensuring high-quality annotations. Specifically, only those audio files receiving unanimous agreement on emotion labels were included in the dataset, resulting in a selection of 9000 files out of the initial 13,000 sent for expert evaluation.

The distribution of emotions within the dataset was carefully considered, with 50% of the files selected for neutral emotions, 10% for sad, 18% for happy, and 22% for angry before augmentation. After applying augmentation techniques, which expanded the dataset by 70%, the emotional distribution was adjusted to 29.41% for neutral emotions, 23.5% for sad, 21.1% for happy, and 25.88% for angry.

Spectrograms, providing visual representations of the frequency spectrum variations over time, were extracted for all utterances using PRAAT software. Each spectrogram corresponded to a single emotion, forming the input data for the subsequent CNN model.

**Figure 10:**
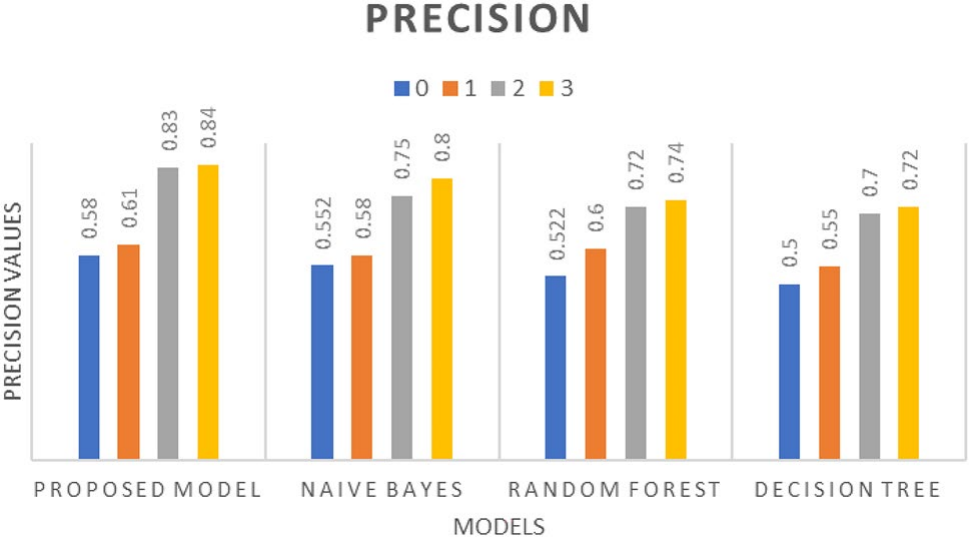
Precision w.r.t. Proposed model, Naïve Bayes, Random Forest, and Decision Tree having ‘0’ as Angry, ‘1’ as Happy, ‘2’ as Neutral, and ‘3’ as Sad.

**Figure 11:**
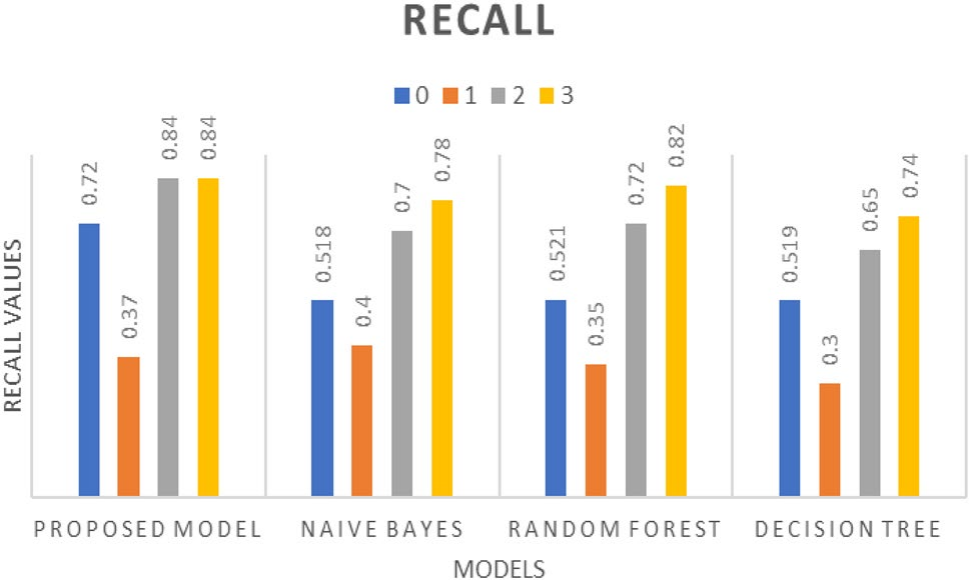
Recall w.r.t. Proposed model, Naïve Bayes, Random Forest, and Decision Tree having ‘0’ as Angry, ‘1’ as Happy, ‘2’ as Neutral, and ‘3’ as Sad.

**Figure 12:**
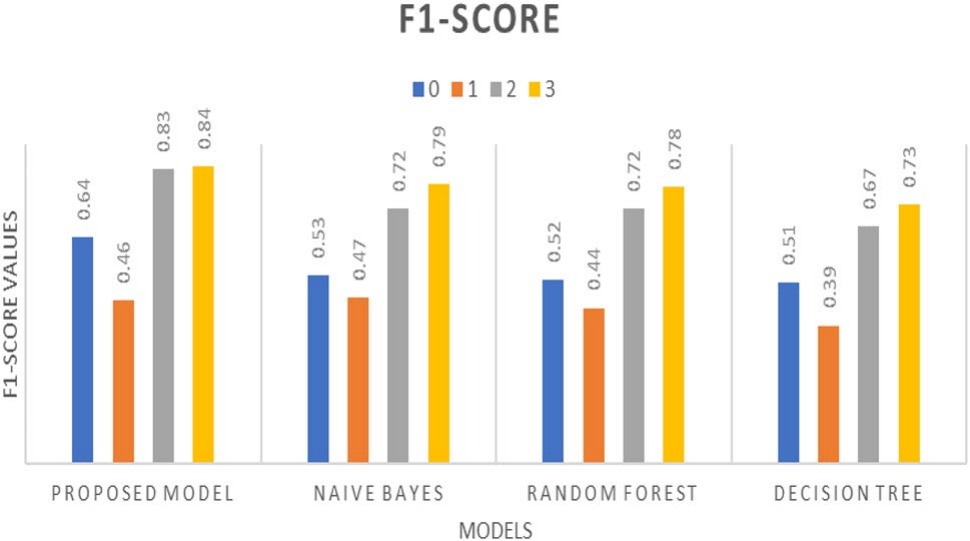
F1-Score w.r.t. Proposed model, Naïve Bayes, Random Forest, and Decision Tree having ‘0’ as Angry, ‘1’ as Happy, ‘2’ as Neutral, and ‘3’ as Sad.

The CNN model was then trained using the spectrogram data and evaluated for various performance metrics, including precision, recall, F1-score, and accuracy. The results, illustrated in Figs. 10, 11, and 12, demonstrate the effectiveness of the proposed algorithm. Notably, the CNN-based approach achieved an accuracy of 69%, outperforming traditional machine learning algorithms such as random forest (61%), decision tree (52%), and Naïve Bayes (49%) as described in Table 3. It has been observed that despite using a deep learning model the achieved accuracy is 69% because of the language model dependency. Punjabi speech poses unique challenges for emotion recognition compared to other languages such as regional accents, dialects, or cultural nuances in expressing emotions could impact the model's performance.

**Table 3:** Comparison of three classifiers with the proposed model in terms of accuracy.

PEMO dataset	Proposed method	Random forest	Decision tree	Naïve Bayes
Accuracy (%)	69	61	52	49

Overall, the experiment successfully demonstrates the efficacy of utilizing deep CNNs for SER in the Punjabi language, showcasing superior performance compared to conventional machine learning techniques. These findings hold promise for applications in sentiment analysis, customer service, and human–computer interaction within Punjabi-speaking communities.

## Conclusion and future work

Emotions play an important role in the day-to-day life of humans. To make human–computer interaction a natural process, automatic speech-emotion recognition is very important. Emotions can be recognized from various modalities like speech, text, facial expressions, etc. However, speech is the most common way through which humans communicate. Speech modality is used in the proposed work to recognize emotions. Moreover, the major difficulty in recognizing the features is in classifying the emotions. There are two types of features, namely, low-level features, and high-level features. In the proposed methodology, high-level features are used whereas previous studies worked on low-level features. The CNN network with high-level features is used to get better performance. These features are extracted from the novel dataset created manually using multimedia and sites in the Punjabi language. This dataset contains audio files and is converted into spectrograms. Afterward, the spectrograms are fed into the CNN model, to classify the audio files into four categories namely, sad, happy, angry, and neutral.

To evaluate the proposed model, four parameters namely, precision, recall, F1-score, and accuracy have been selected. It has been concluded that the proposed methodology outperforms decision trees, naive Bayes, and random forests. This is a novel work as very little research has been conducted in this area using the Punjabi language.

Therefore, this research will open new avenues for upcoming researchers. For future work, this area can be expanded in three ways.

- In the proposed work, 9000 audio files were collected to recognize the emotions in Punjabi language. To have a high impact, there is a need to create a large dataset by increasing the count of audio files. This will help to get improved and optimized results.
- Only one modality has been implemented in the proposed work. The work can be extended using text, facial expressions, and electroencephalogram (EEG).
- In the proposed work, the implementation has been done in one language. In the future, this can be compared with more than one language to check the dependency of one language on another. This will increase the effectiveness of human–machine interaction.

## Data Availability

Data is available from the authors upon reasonable request from the corresponding author.
