# Author Correction: Emotion recognition for human–computer interaction using high-level descriptors

**DOI:** 10.1038/s41598-024-64365-1

**Published:** 2024-06-18

**Authors:** Chaitanya Singla, Sukhdev Singh, Preeti Sharma, Nitin Mittal, Fikreselam Gared

**Affiliations:** 1 Chitkara University Institute of Engineering and Technology, Chitkara University, Punjab, India; 2 Department of Computer Science, Multani Mal Modi College, Patiala, Punjab India; 3 Skill Faculty of Engineering and Technology, Shri Vishwakarma Skill University, Palwal, Haryana 121102 India; 4 Faculty of Electrical and Computer Engineering, Bahir Dar University, Bahir Dar, Ethiopia

Correction to: *Scientific Reports* 10.1038/s41598-024-59294-y, published online 27 May 2024

In the original version of this Article Nitin Mittal was incorrectly affiliated with ‘Department of Computer Engineering and Application, GLA University, Mathura, Uttar Pradesh, India’. The correct affiliation is listed below:

Skill Faculty of Engineering and Technology, Shri Vishwakarma Skill University, Palwal, Haryana 121102, India

The original Article has been corrected.

